# A contralateral wing stabilizes a hovering hawkmoth under a lateral gust

**DOI:** 10.1038/s41598-019-53625-0

**Published:** 2019-11-22

**Authors:** Jong-Seob Han, Jae-Hung Han

**Affiliations:** 1 Department of Aerospace Engineering, Korea Advanced Institute of Science and Technology (KAIST), 291 Daehak-ro, Daejeon, Republic of Korea; 2 Present Address: Technical University of Munich, Garching, Germany

**Keywords:** Aerospace engineering, Biomimetics, Aerospace engineering, Biomimetics

## Abstract

Previous analysis on the lateral stability of hovering insects, which reported a destabilizing roll moment due to a lateral gust, has relied on the results of a single wing without considering a presence of the contralateral wing (wing-wing interaction). Here, we investigated the presence of the contralateral wing on the aerodynamic and flight dynamic characteristics of a hovering hawkmoth under a lateral gust. By employing a dynamically scaled-up mechanical model and a servo-driven towing system installed in a water tank, we found that the presence of the contralateral wing plays a significant role in the lateral static stability. The contralateral wing mitigated an excessive aerodynamic force on the wing at the leeward side, thereby providing a negative roll moment to the body. Digital particle image velocimetry revealed an attenuated vortical system of the leading-edge vortex. An excessive effective angle of attack in the single wing case, which was caused by the root vortex of previous half stroke, was reduced by a downwash of the contralateral wing. The contralateral wing also relocated a neutral point in close proximity to the wing hinge points above the actual center of gravity, providing a practical static margin to a hovering hawkmoth.

## Introduction

Continuous back-and-forth two flapping wings of insects, which change inflow direction periodically, give rise to unique flight dynamic characteristics. For example, hovering insects can earn ubiquitous passive damping called flapping counterforce/torque^1–4^. During yaw turns, the angular velocity of the body results in asymmetries of inflow velocities on two flapping wings, supplying yaw damping moment on the body that counteracts to the yaw turns. The disturbance from the indirect axis, on the other hand, remains as an element in destabilizing hovering insects^5^. This instability during hovering has been commonly found in various species of insects including a bumblebee, hawkmoth, fruit fly, hoverfly, crane fly, drone fly, and stalk-eyed fly^6,7^.

An instability (an unstable slow divergence mode) in a lateral direction was known to stem from the change in the roll moment due to the perturbation of a lateral gust^8–10^. This indicates that insects should generate finely-tuned strong control force to mediate the rolling motion when insects intend to voluntarily move sideways, or to overcome a lateral gust thereby maintaining the hovering state. According to Sun and his colleagues^5,11^, the gust running sideways brings out two significant aerodynamic outcomes on flapping wings, i.e., (1) an effect of ‘changing-relative-velocity’ and (2) an effect of ‘changing-axial-velocity in leading-edge vortex (LEV)’. The changing-relative-velocity is associated with a resultant inflow heading perpendicularly to the wing surface, which is determined by the combination of the feathering and gust speeds, implying that the effect would be nearly canceled out by its bilateral symmetry with the contralateral wing. The effect of changing-axial-velocity in LEV, on the other hand, directly intensifies the LEV thereby augmenting aerodynamic lift on the wing at the leeward side than that in the windward side. They attributed such reinforcement to the instability in the lateral direction; the imbalance leads to the positive roll moment derivative and results in the unstable divergence mode.

One critical feature under lateral gust is that a presence of the contralateral wing (wing-wing interaction) should be considered^12,13^, in contrast to that in forward flight, in which most of the downwash and/or wake gently slipped out over the abdomen. Let us suppose the gust is running from the right side. This gust should pass the flow field that had been influenced by the right wing before reaching the left wing. Such a distorted gust could then directly impede the spanwise flux of LEV and aerodynamic benefit on the left wing, and thereby influence the lateral flight stability of the flapping-wing system. Indeed, an integrated computational framework including two wings and body, encompassing the effect of the contralateral wing^14^, clearly exhibited the initial static stability of a hovering fruit fly. The model fruit fly eventually lost its orientation because of the dynamic instability, but the static stability was absolutely sufficient for a nervous feedback loop (the transition period becoming unstable from the equilibrium state was much longer than a time delay from sensory systems to motor neurons). This was contrary to the other studies aforementioned; however, they failed to explain where the static stability came from. A recent study of Han *et al*.^13^ speculated that the presence of the contralateral wing and the wing-wing interaction could bring out a favorable effect on the lateral static stability. Their study, which was weighted to the fundamental aerodynamic characteristics with simplified variables, rather asks further investigations with suitable morphological parameters of a living insect.

Here, we employed a dynamically scaled-up mechanical model with a servo-driven towing tank, which is able to provide accurate data of aerodynamic loads and flow behaviors under a lateral gust. The model generating the back-and-forth motion of a hawkmoth *Manduca sexta* in hover^15^ was traveled by the towing system with designated gust conditions. We measured time-varying aerodynamic force and moment, and converted to that on the body-fixed coordinate system by combining with the morphological parameters of a hawkmoth^16,17^. By comparing the cases which considered the contralateral wing or not, we found that the presence of the contralateral wing plays a significant role in the lateral stability.

## Results

### Effect of the contralateral wing in hover

**Figure 1:**
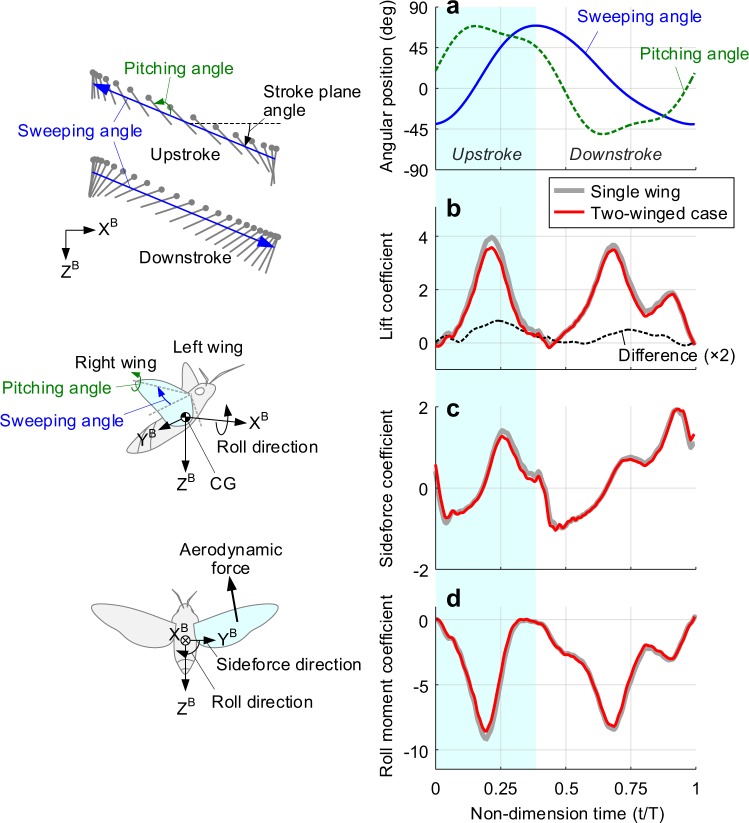
Time-varying wingbeat motion and aerodynamic forces and moment during a single wingbeat cycle in hover. (**a**) Sweeping (blue) and pitching (green) angles of the right wing; angle deviation from the stroke plane was not considered in this study. (**b**–**d**) Aerodynamic coefficients on the body. (**b**) Lift (vertical direction). **c** Sideforce (to the right). (**d**) Aerodynamic roll moment (along the X-axis). Gray thick line and red thin line denote the single wing case and two-winged case, respectively. The difference is multiplied by two for the sake of clarity. The shaded area from t/T = 0 to t/T~0.4 indicates the upstroke.

Most previous studies on the lateral stability^8^ had been undertaken in an assumption of ‘insignificant wing-wing interaction’. Two wings on the thorax of a hawkmoth, however, are in close proximity to each other; the distance between the two hinges only is less than 20% of the wingtip-to-wingtip distance^18^. Thus, we first compared the aerodynamic characteristics in the single wing case to the two-winged cases with the mechanical model. Figure 1a shows a wingbeat motion in a single wingbeat cycle T. We applied a stroke plane angle (the inclined angle from the horizontal plane) and time-varying sweeping and pitching angles of a living hawkmoth^15^. The angle deviation from the stroke plane, which is rarely changed in hover, was not adopted in this case. Figure 1b–d show aerodynamic coefficients of the lift, sideforce, and roll moment. These were measured on the right wing with a sensor installed at the wingbase, and then transformed to the body-fixed coordinate system, which is denoted with a superscript B (refer to the schematics in Fig. 1). No gust is applied here.

In the lift curves, two peaks were found near t/T = 0.22 and t/T = 0.69, when the wing reached the maximum stroke velocity (Fig. 1a). The sufficient pitching-up velocity in late downstroke near t/T = 0.90 made a third peak conspicuous in contrast to that in the upstroke. A sideforce appeared as negative and positive forces depending on the direction of the wing surface in each up- and downstroke, made a force fluctuation on the Y^B^-axis twice. The roll moment showed a nearly analogous variation with the lift force because it was mainly produced by the aerodynamic lift. The sideforce and roll moment, however, will be eventually ineffective on the body, because the force and moment on the left wing counteract against that on the right wing.

Note the values when the contralateral wing (the left wing here) is installed (red thick lines). Not only the lift, but the other two components always marked lower values in the entire wingbeat cycle. The differences between them (thick dashed lines) shows two peaks at the middle of up- and downstroke without noticeable valleys, clearly support such a global influence of the contralateral wing on the aerodynamic characteristics. The levels of decrements in the lift, sideforce, and roll moment were 9.3%, 12.4%, and 4.2%, respectively; these clearly indicate that the effect of the contralateral wing is not negligible at all.

A recent parametric study by Han *et al*.^12^ revealed that two rectangular flapping wings with an aspect ratio of four always result in lower aerodynamic performance than that of the single wing combination. This mainly stems from attenuated angles of attack near each wing base caused by the interaction of two downwashes. They demonstrated that the level of reduction appeared up to 4.5%, and inferred that the shape of wings of living insects, which had wider wing area near the wing base with a shorter length, would accelerate such reduction. The reduction of 9.3% in the lift in this study, which is more than twice of that in the previous study^12^, does not only support above inference but further informs us that the contralateral wing must be considered in the lateral stability analysis.

### Effect of the contralateral wing under a lateral gust

**Figure 2:**
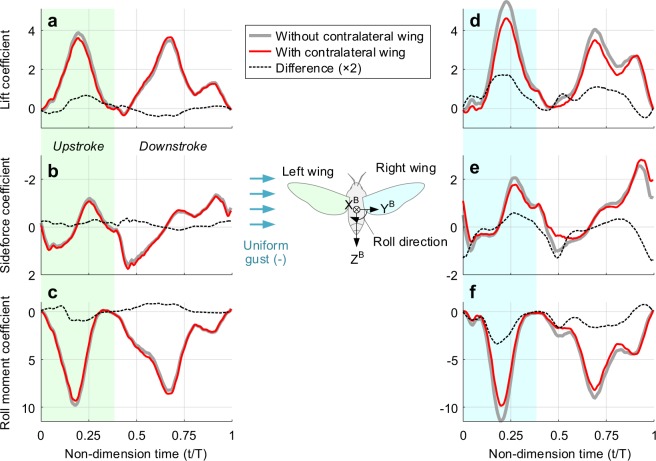
Time-varying aerodynamic forces and moment on the body under the lateral gust. The gust ran from the left with a speed of 13.3% of the mean wingtip speed. (**a**–**c**) Aerodynamic coefficients on the left wing; the y-axes in sideforce and roll moment coefficients were inverted within the same scale of that on the right wing to compare the time-historical changes. (**d**–**f**) Aerodynamic coefficients on the right wing. Gray thick line, red thin line, and black dashed line denote the single wing case, two-winged case, and these differences. The differences were enlarged two times for better visibility. The shaded area from t/T = 0 to t/T ~ 0.4 indicates the upstroke.

Figure 2 shows the aerodynamic coefficients under a lateral gust, which ran with 13.3% of the mean wingtip velocity. The gust ran from the left, so the lift augmentation aforementioned^5^ must show up on the right wing. Let us see the left wing first (Fig. 2a–c). Regardless of the presence of the contralateral wing, all three components governing the lateral motion did not change significantly. There are little fluctuations in the differences (thick dashed lines), which appear to be caused by slightly biased mean sweeping angle to the dorsal part as Han *et al*.^12^ already pointed out (the wings moved from −40.7 to 72.4 in the sweeping angle; biased 15.7 deg from the Y^B^-axis), but cycle-averaged values nearly remain zero. This implies that the lateral gust running from the wingtip did not bring on significant influences on the wing at the windward side. This is reasonable because the gust in this circumstance does not face any obstacles before reaching the wing of interest.

On the right wing, we first found that the absence of the left wing resulted in excessive reinforcements in both the lift and roll moment with the lateral gust (Fig. 2d–f). The first peak of C_L_ in the upstroke scored ~5.5, which is 41% higher than that of the left wing. The cycle-averaged values of the lift and roll moment were also 42% and 11% stronger than that of the left wing, fully sufficient to bring out a destabilizing roll moment of the body (positive roll moment derivative). This is in line with the previous analysis that the lift augmentation on the far side of the wing is the primary source to destabilize the hovering insect under the lateral gust^5^.

Such augmentations, however, completely disappeared when the left wing was considered. As shown in red curves in Fig. 2d–f, the lift always marked lower values except a particularly tight instance where the wings had a ventral stroke reversal (0.9 < t/T < 1.0). Two major peaks in each stroke were reduced by 18.4% and 15.6%, respectively, resulted in 16.3% lower value in the cycle-averaged lift. The sideforce also experienced a considerable change. This mainly stemmed from the reduced wing-wake interaction that had appeared right after each stroke reversal (t/T ~ 0.48 and ~0.98); this brought out 16.4% higher value in the entire wingbeat cycle. The cycle-averaged roll moment of the right wing, which showed a similar tendency to the lift in the timeline, was also reduced by 15.0%. All these clearly showed that the additional aerodynamic force owing to the lateral gust is practically mitigated by the contralateral wing.

**Figure 3:**
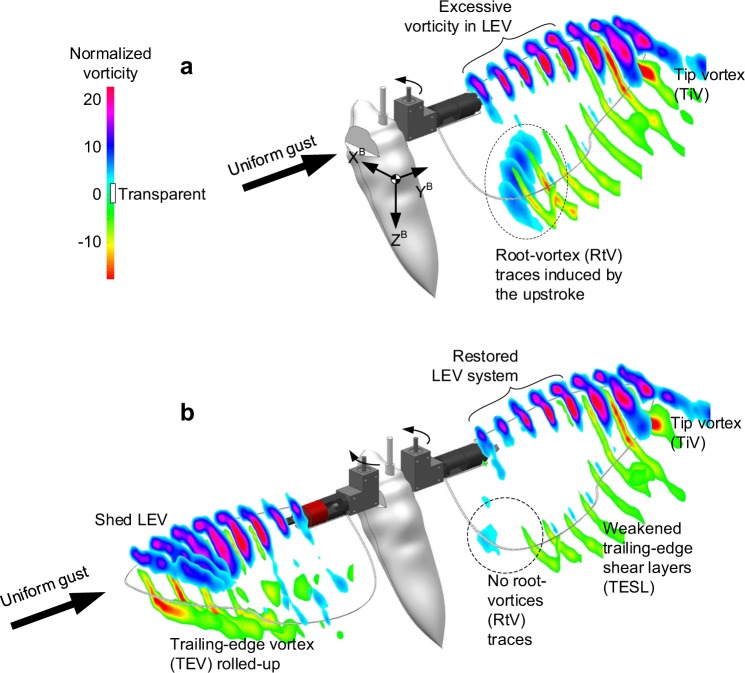
Vorticity distributions at t/T = 0.69. The lateral gust uniformly hit the model with 13% of the mean wingtip velocity from the left. The vorticities were extracted from the velocity fields in two-dimensional DPIV, and were normalized by the mean chord length and mean wingtip velocity. The sections started from 0.2*b* to 0.9*b* at an interval of 0.1*b*, where *b* denote the spanwise length. (**a**) Right wing only. (**b**) Two-winged case. The body model was attached in both cases.

Figure 3 shows the phase-averaged vorticity fields at t/T = 0.69, where the second peak was developed (refer to the Methods II for more details). As expected, we found an excessive LEV system on the right wing when the contralateral wing was absent (Fig. 3a). The LEV system continued to 0.8*b* from the wingroot, and the tip vortex (TiV) arose only at 0.9*b* with a rolled-up trailing-edge vortex (TEV). At the inboard sections from 0.2*b* to 0.6*b*, the excessive vorticities in LEV were uniformly distributed. This formed a cylindrical shape rather than a conical shape that had been reported in numerous previous studies in hover (refer to^19–23^ to see the LEV structure at a glance). These all clearly indicate the effect of the lateral gust on the LEV and additional aerodynamic force.

The presence of the contralateral wing reduced the LEV system on the right wing (Fig. 3b). It more clearly appeared near the wingbase from 0.2*b* to 0.6*b*; the LEV system was completely restored to the conical shape. One noteworthy feature is that strong wingroot vortex (RtV) traces of the previous half stroke in the single wing case, which covered up to the 0.5*b* from the wingroot, disappeared when the contralateral wing was present. Accordingly, trailing-edge shear-layers (TESLs) near the wing root were also attenuated. Han *et al*.^13^ explained that such RtV traces can only be sustained in the single wing case; the presence of the contralateral wing gives rise to an interaction of two individual downwashes, which can wash down the RtV traces with attraction to each other. They further revealed that the interaction of two downwashes resulted in lower effective angles of attack near the wing base, and attenuated aerodynamic forces. The DPIV results in this study clarify that the interaction in the previous study is still valid even at a hovering hawkmoth.

**Figure 4:**
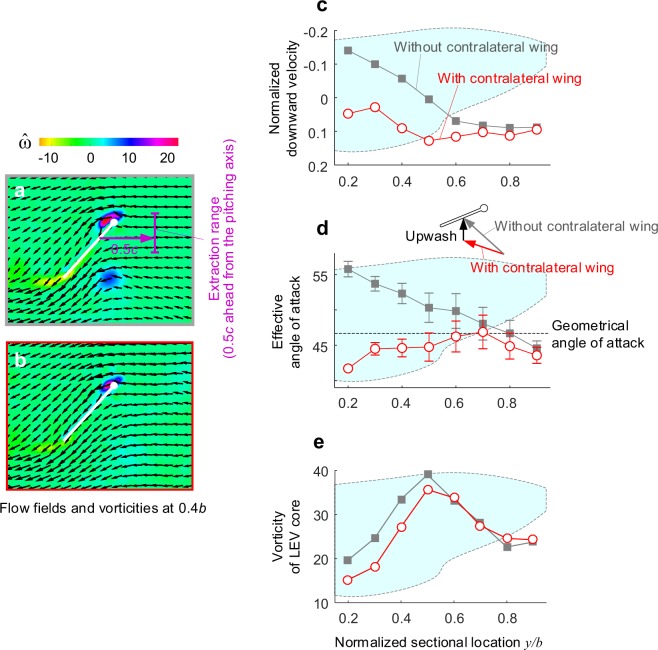
Detailed flow fields and some flow quantities at 0.4*b* of the right wing. Gray squares and red circles with each line denote the case of the right wing only and two-winged case, respectively. (**a**,**b**) Velocity and vorticity distributions on the right wing. (**a**) The right wing only. (**b**) Two-winged case. Gray and red boundaries in each field denote each configuration. (**c**) Normalized downward velocity. (**d**) Effective angles of attack. (**c**,**d**) Were extracted 0.5c ahead of the pitching axis (the purple line in (**a**); the vertical range was ± 0.2c about the pivot point. (**e**) The maximum vorticity of the LEV in each cross-section.

Figure 4a,b describe the detailed flow fields obtained at 0.4*b* of the right wing which are already provided in Fig. 3. In the single wing case, the strong RtV trace created the upwash with distorting the flow field (Fig. 4a). The presence of the contralateral wing removed the RtV trace (Fig. 4b). The inflow became less distorted. The downward velocity increased (Fig. 4c), and the effective angles of attack decreased (Fig. 4d). These appeared strongly near the wingbase, and gradually lessened up to 0.6*b*, eventually brought out quite lower vorticities on inboard cross-sections (Fig. 4e). These are all in line with the interpretation above.

### Effect of the contralateral wing on the flight stability

**Figure 5:**
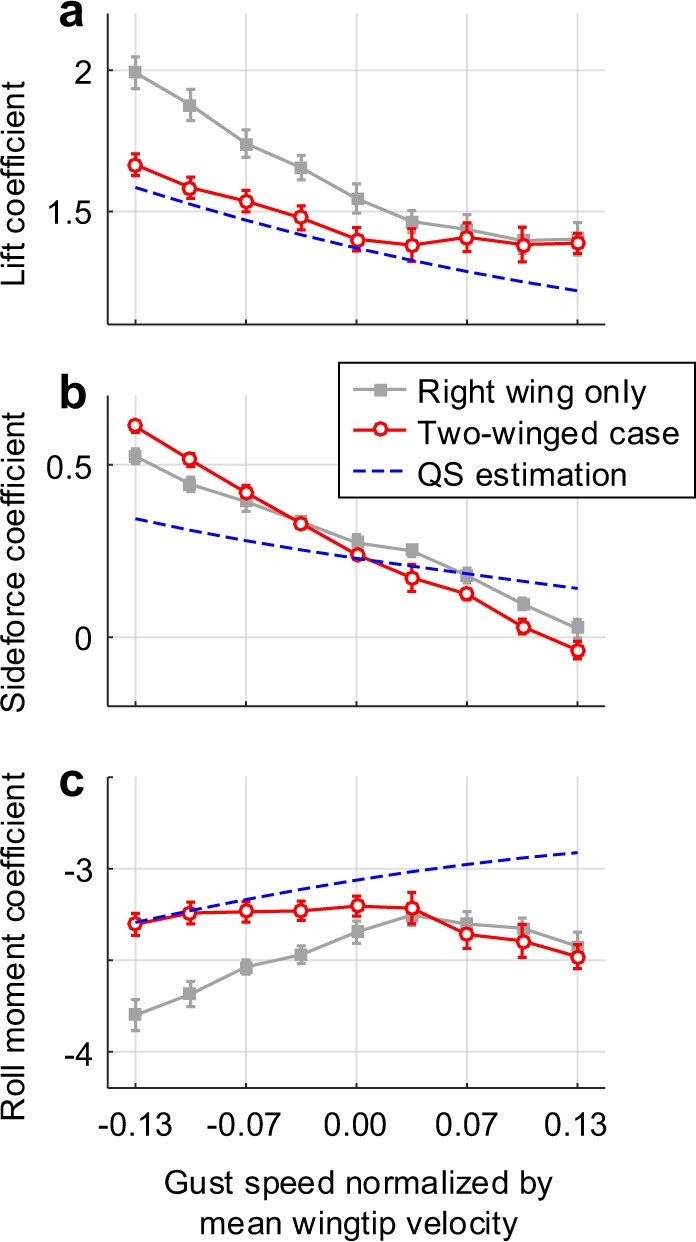
Cycle-averaged aerodynamic coefficients on the right wing with respect to the gust speeds. Gray squares and red circles with each line denote the case of the right wing only and two-winged case, respectively. Blue dashed lines indicates the estimated values via the quasi-steady aerodynamic model^25–27^. (**a**) Mean aerodynamic lifts. (**b**) Sideforces. (**c**) Roll moments on the body.

Figure 5 shows the cycle-averaged forces and moment on the right wing with respect to the gust speeds. The presence of the contralateral wing certainly mitigated the excessive aerodynamic lift in the single wing case (Fig. 5a). This particularly appeared under the negative lateral gust; the lift here was rather closer to the quasi-steady estimation, which does not take any effect of the axial flux into account (as discussed later). The contralateral wing also resulted in a larger decline rate in the sideforce (Fig. 5b), and a substantial reduction in the roll moment magnitude at the negative lateral gust (Fig. 5c). Smaller magnitude in roll moment than that of the single wing case further suggests potential stability in the roll direction.

**Figure 6:**
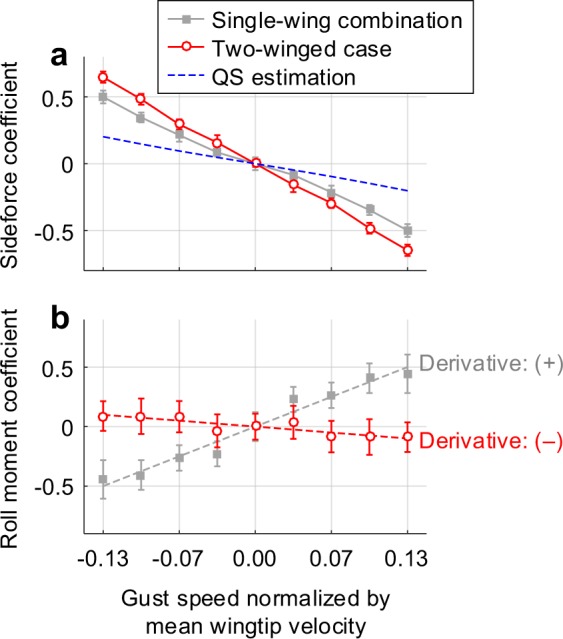
Aerodynamic force and moment of two flapping wings on the body. Gray squares and red circles with each line denote the single wing combination and two-winged case, respectively. (**a)** Sideforces on the body. (**b)** Roll moment on the body; The dashed lines are the regression curves in each case.

Based on the mean values above, we compared the sideforce and roll moment on the body in the actual case (with the contralateral wing) to the single wing combination (Fig. 6). Here, the single wing combination denotes the summation of aerodynamic loads of the isolated left wing and the isolated right wing, and the force and moment on the left wing were estimated with the bilateral symmetry about the longitudinal plane. The values in Fig. 6 are applicable for flight stability analysis, despite the fact that a hovering hawkmoth is a nonlinear time-variant dynamic system with a periodic aerodynamic force/moment^5^. This is because a hawkmoth body has a relatively heavier weight and lower natural frequency than the wingbeat frequency like the other hovering insects aforementioned, implying that at least several wingbeats are required for a sufficient change in the body orientation. This wingbeat-cycle-averaging approach has been validated with numerical solutions of the complete equations of motion coupled with the Navier-Stokes equations^7^. The rare change in the wingbeat kinematics during an evasive maneuver^3^ and the relatively long latency from sensory neurons to muscles from strong visual stimuli (the reaction time of a steering muscle is up to 100 ms, which corresponds to ~2.5 wingbeats^24^) also supports the wingbeat-cycle-averaging approach adopted.

In the longitudinal direction, a negative pitching moment derivative indicates static stability because it produces counteract pitching moment against an arbitrary orientation of a vehicle. Exactly the same principle is applicable to the stability analyses on the sideways and roll directions. The inertial force/moment caused by the wingbeat motion are negligible here, because both wings had an identical motion profile so the cycle-averaged inertial force/moment became zero in hover. Figure 6a shows that the sideforce had larger decline rate than that of the single wing combination as well as the quasi-steady estimation. This suggests a stronger flapping-counter force (FCF) acting on a living hawkmoth than that of the theoretically derived FCF with the quasi-steady assumption, owing to the presence of the contralateral wing. In terms of the roll moment (Fig. 6b), the presence of the contralateral wing inverted the roll moment derivative to negative (stabilize). This shows that the contralateral wing can bring out the lateral static stability in the roll direction under the lateral gust.

## Discussion

### Where does the excessive aerodynamic force come from

Our analysis on the flow structures using the DPIV (Fig. 4) indicated that the lift augmentation in the single wing case under the negative lateral gust (running from the wingroot) could be explained by the presence of the RtV at the inboard sections and the increase in the effective angles of attack, aside ‘the changing axial-velocity in LEV’^5^; the RtV here plays a role to pull the inflow and to add the upward component into the freestream (upwash) as shown in Fig. 4d, similar to the wing-wake interaction (refer to^23^ for the detailed vortex formations of the wing-wake interaction). Indeed, our simple arithmetic using the effective angle of attack and the quasi-steady concept showed 18.2% higher than that of the original estimation at this instance (here, we used the lift coefficient in the translational model which is the function of the angle of attack only; refer to the Methods IV and^25–27^ for details). This value was also 11.9% higher than that of the two-winged case; the value corresponds to 76.3% of the difference in the two direct measurements (15.6%). These suggest that most augmentation could stem from the change in the effective angle of attack, although the stretched LEV system near the outboard of the wing sections indicates the presence of ‘the changing axial-velocity in LEV’.

### Contralateral wing mitigates the excessive force

The analysis further showed that except the reinforced LEV system from the wingbase to 0.6*b*, the distributions on the right wing in the two-winged case appear to be similar to the single wing case. In both cases, two individual LEV systems were eventually stabilized throughout the leading edge, and only small rolled-up TEV and TiV appeared at 0.9*b* regardless of the presence of the contralateral wing. This is rather distinct from that in hover in which the TiV are developed from ~0.7*b*^13^, implying that the contralateral wing (left wing) did not directly interrupt the additional flux along the radial direction on the wing of interest (right wing).

Instead, we found notable interest in the effective angles of attack, which were completely restored to the geometrical values as shown in Fig. 4b. Accordingly, the lift became closer to that of the quasi-steady estimation (Fig. 5a); this also indirectly supports that the axial flux was not the key source for the additional aerodynamic forces. The vorticities of LEV near the wingbase were also directly attenuated. These were apparent from the wing base to 0.6*b*, which showed up to 21.1% lower values. The mean vorticity of the LEV system was, therefore, 9.2% lower than that in the single wing case (Fig. 4c).

It should be noted that the RtV traces of the previous half stroke, which had encroached into 0.5*b* in the single wing case, disappeared with the contralateral wing. This clearly indicates the existence of downward flux that is sufficient to modify the inflow direction with washing down the RtV traces, as Han *et al*.^12,13^ already found. This suggests that the downwash induced by the contralateral wing is the crucial source both to decline the inflow direction and to reduce the effective angles of attack near the wing base (Fig. 4b), thereby restoring both the LEV and the aerodynamic lift from the unusual augmentations, even in a wing motion of living hawkmoth. If a hawkmoth is moving to the left from hovering, for example, then the right wing will be continuously submerged in the downwash created by the left wing thereby producing usual aerodynamic force and moment.

One interesting point is that such downwash behavior is also likely to be acceptable for other insects adept at hovering. This is because all the insects and a hummingbird produces massive downward flux to stay aloft with a similar wingbeat motion, i.e., almost horizontal stroke plane with an excessive angle of attack beyond the stall angle of an airfoil. A similar amount of the maximum lift coefficients, which did not change much with respect to the flight regime of the Reynolds number^28,29^, clearly reflects each downward flux corresponding to the weight of each species, and strengthens our inference.

### Forces and moment in practice versus the quasi-steady estimations

The quasi-steady model used here includes all three components of the translational, rotational, and added mass contribution (refer to the Methods IV for details). However, it always underestimated the aerodynamic forces and moment under lateral gust (Fig. 5). This mainly comes from certain instances exceeded the quasi-steady assumption (e.g., near each stroke reversal)^25–27^. Accordingly, these result in somewhat different cycle-averaged values in each component, as well as those derivatives pertaining to the lateral motion. The fact that the trim search *via* the quasi-steady model yielded a higher wingbeat frequency than the observation^17^, is a conspicuous example of such drawback of the model.

Another noticeable feature is that the difference of 12.9% in the aerodynamic lift was much higher than that of the previous studies which only left up to ~7%^26^. This seems to be caused by the slightly more complicated motion profiles of a living hawkmoth than the simplified one^15^, suggesting that a hawkmoth would employ the unsteady flow more proactively for maintaining the hovering state. Two dominant unsteady features found in the direct simulation for a living fruit fly^30,31^, indirectly support our inference. This further indicates that the evaluation with the quasi-steady aerodynamic model cannot guarantee the accurate interpretation at least in the lateral motion. Several papers suggested the static stability in the lateral motion^17,32^ were also based on a coincidence in the effective angle of attack that originally resulted from the downwash of the contralateral wing.

### Contralateral wing stabilizes hovering insects

**Figure 7:**
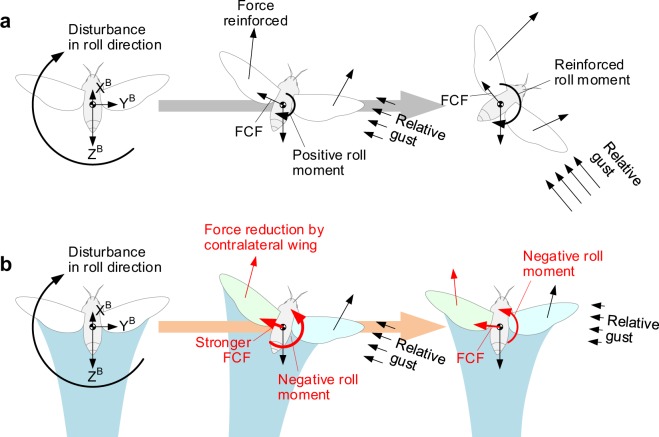
Back views of two different responses to the disturbance in roll direction, predicted by the roll moment derivatives. (**a**) Single-wing combination. (**b**) Two-winged case. Shaded region below the wings signifies the downwash field produced by two flapping wings. Red arrows denote the influences by the presence of the contralateral wing.

According to the previous interpretations, which had adopted the single-wing aerodynamics^5,8,9^, the perturbation in the roll direction makes the hawkmoth unstable. As shown in Fig. 7a, the sideslip and relative gust due to the perturbation gradually augments the aerodynamic force on the left wing which is located at the leeward side. This accelerates the change in roll angle with increasing gust speed (or increasing sideslip speed).

Figure 7b explains how the contralateral wing stabilizes a hovering hawkmoth in reality. Let us suppose the present distubance creates a positive roll moment. The lift then slightly tilts to the right, the insect slipped to the right and faced with the lateral gust coming from the right within several wingbeat cycles (as discussed earlier, the body weight is relatively heavy and wingbeat frequency is much higher than that of the natural frequency of the body). The left wing is therefore slightly submerged in the downwash field of the right wing (the contralateral wing), less aerodynamic lift and moment is produced on the left wing, thereby generating a stabilizing roll moment on the body; the body is eventually recovered to the initial attitude without control. Here, the stronger flapping-counter force^1–4^ may accelerate the recovery by quickly weakening the gust speed. The location of CG^16,17^ is also essential to generate the negative roll moment because the reduction in the aerodynamic moment caused by the contralateral wing is insufficient to invert the roll moment on the body. The other roll moment component produced by the sideforce and the distance from the CG to the wing hinges completed the negative roll moment.

**Figure 8:**
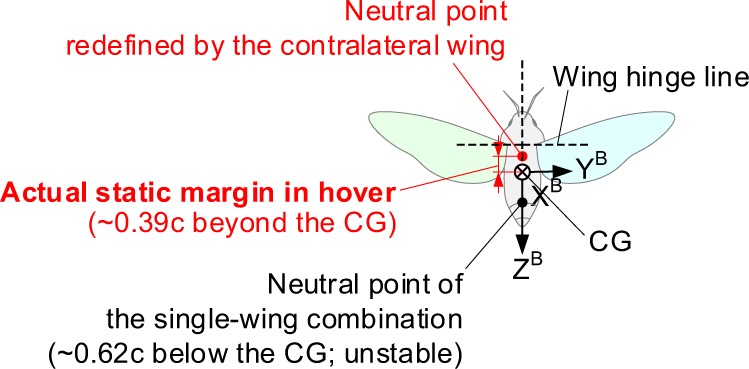
Neutral points (the artificial CG points at which the model is neutralized) and a static margin of the model hawkmoth (back view). Red colors denote the actual case (with the contralateral wing).

One useful concept to analyze the flight dynamic characteristics of a fixed-wing vehicle is to find an artificial CG point at which the stability becomes neutral (so-called a neutral point). The stability and controllability of the aircraft can also be assessed by the distance of the CG from the neutral point (a static margin). The CG in front of the neutral point with a certain static margin, for example, stabilizes the aircraft with a negative pitching moment derivative, but it also degrades the maneuverability if the CG is too far ahead from the neutral point. The CG behind the neutral point, on the other hand, destabilizes the vehicle but gives higher controllability^33^. By using the same concept, we analyzed the neutral point and the static margin in the lateral direction as shown in Fig. 8. The neutral point here is defined as a theoretical CG point satisfying marginal lateral stability. Compared to the longitudinal stability analysis, in which the CG should lie ahead of the neutral point to acquire the static stability, the CG here should be located below the neutral point for the lateral static stability similar to a pendulum.

With this concept, we found that the reduction in the aerodynamic lift due to the contralateral wing dramatically elevates the neutral point far beyond the actual CG of a hawkmoth. This does not only imply that a hovering hawkmoth inherently possesses the initial static stability in the lateral direction, but also the contralateral wing allows the CG in close proximity to the wing hinge point. This allows pulling down of the stroke plane or up of the abdomen (CG) to a certain level in order to manipulate their flight^28^ without losing the lateral static stability. In case of bio-inspired drones^34,35^, the CG can be designed closer to the wing hinge point than that of the estimation from the previous CFD analysis because of the presence of the contralateral wing.

## Methods

All the apparatuses employed in this study had been specified in the authors’ papers published elsewhere; certain key status is provided here (refer to^13^ for more details).

### Dynamically scaled-up mechanical model and servo-driven towing tank

**Figure 9:**
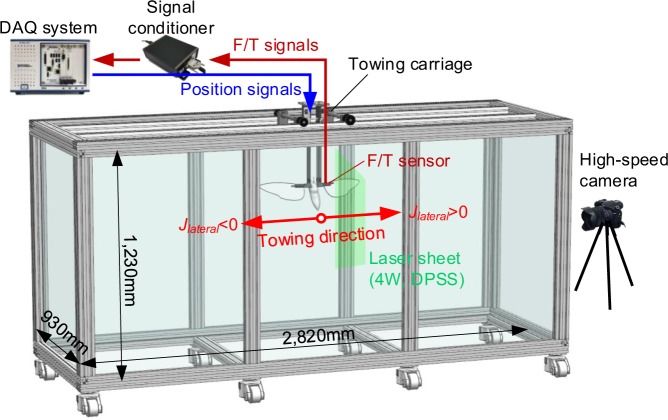
A schematic of the experimental apparatus.

Figure 9 shows the schematic of the experimental apparatus. The hawkmoth-like mechanical model had been scaled up approximately five times of the target hawkmoth, *Manduca sexta*^13^. Each wing, which had been made of 2-mm acrylic plate for sufficient rigidity, has a spanwise length *b* of 250 mm and an aspect ratio of 3.09. The root-to-root distance between two wings was 0.18 times of the tip-to-tip distance, which is similar to the hawkmoth^18^. The body was fabricated by 3d-printer, which has a simplified shape of a hawkmoth^36^. This was also scaled up about five times, and has a length of 0.81*b*^16^. Four servo motors installed on the model had been in charge of creating wingbeat motion of the hovering hawkmoth^15^ with a wingbeat frequency of 0.125 Hz; corresponding Reynolds number became ~1.0 × 10^4^ and it was equivalent to that of the hawkmoth in hover. The water tank has a size of 2.82 m (L) × 0.93 m (W) × 1.23 m (H); the length was 4.6 times larger than the tip-to-tip distance of the model hawkmoth. One additional servo motor on a towing system of a water tank had been employed to continuously drift the model along the longitudinal direction of the tank. The model hawkmoth thereby had consistently experienced a uniform lateral gust during the measurement. The measurement uncertainties of the force and moment had been calculated as ±2.02% (±0.012 N) and ±0.97% (±0.055N-mm), where the percentages are based on the maximum amplitude in our measurement.

### Force/moment/DPIV measurement

6-axis force/moment sensor (Nano17-IP68, ATI Industrial Automation) connected to a DAQ system (PCIe-6361, National Instruments) was installed on the right wing to collect the time-varying aerodynamic force/moment. The force/moment were then relocated to the center of gravity of the body with a coordinate transformation matrix, which is the function of the wing kinematics and the vectors designating the sensing point from the center of gravity (as discussed later). Four individual conditions including a right wing only, a right wing and a body, two wings, and two wings with body had been considered with seven different lateral gusts; the results from the effect of the body were omitted in this paper because of its negligible effectiveness. Note that we did not directly collect the aerodynamic force/moment on the left wing. Instead, it had been obtained by converting the values acting on the right wing with its bisymmetry about the longitudinal plane (the aerodynamic force/moment on the left wing should be the same of the right wing in the lateral gust running to the opposite direction). In each case, an entire trajectory was composed of (1) five continuous wingbeat motion with moving a sideward, (2) rewinding to the initial point, and (3) resting for recovering the quiescent flow. This was in order to avoid both contaminations from a sudden departure of the wing motion at very beginning and an underdeveloped wake, which nearly disappeared within two wingbeat cycles in hover^12–14^. All the data represented here were extracted from the fifth wing beat cycle. This process had been iterated 21 times to remove random noises. The measurement uncertainty was obtained from the range of wingbeat motion and angular resolutions of the robotic model (360/4096°, XH430, Robotis), a spatial resolution in the command signal (updating interval of 5 ms, 1/1600T), and the resolution of the sensor of 3.13 × 10^−3^ N and 15.6 × 10^−3^ N-mm. These remained within ±2.53% in the lift, ±3.13% in the sideforce, and ±0.87% in the roll moment of the maximum levels of each component, respectively (refer to^13^ for details).

A diode-pumped solid-state laser of four watts and a high-speed camera (RX10-III, Sony) had been employed for the DPIV measurement. The image pairs had been collected when the wing passed the desired temporal/spatial instance in the fifth wingbeat cycle. Here, the locations of interest had been designated by an in-house code with a simultaneously operated high-speed camera *via* a trigger pulse. In each cross-section, a total of 53 phase-averaged image pairs was recorded to extract the mean velocity at t/T = 0.69 in the fifth cycle. Field of view (FOV) was fixed as 5.85c (L) × 3.29c (H) at each cross-section, where c denotes the mean chord length. An open-source DPIV code^37^ was then employed to analyze the pair. An interrogation area of 40 × 40 with overlapping 50% gave us the vector fields of 95 × 53 from the original images which consisted of 1,920 × 1,080 pixels; the spatial resolution became 0.061c × 0.061c. For the reconstruction to 3d space, the other in-house code written in MATLAB® was used. One thing should be highlighted is that the out-of-plane motions of the particles perpendicular to the FOV, which could be accelerated by the lateral gust, were barely observed in this process, similar to the previous study^13^. This was because a relatively thick laser sheet (>3 mm) and sufficiently short intervals in pairs of images (1/250 s; 1/2000 T); this thickness corresponds to ~44 times of the out-of-plane particle displacement (17.0 mm/s), which is induced by the lateral gust with 13.3% of the mean wingtip velocity. The particle density over 19 in each interrogation area also relieved the degradation in the PIV algorithm^38^. The DPIV uncertainty, which was calculated with mean particle image diameter of 4.03 pixels, displacement of 0–2.55, and density in the interrogation area *N* ~ 19, remained the errors of −0.024 ± 0.072; these correspond to −0.94 ± 2.8% of the maximum displacement.

### Converting the force/moment to the CG of a hawkmoth

**Figure 10:**
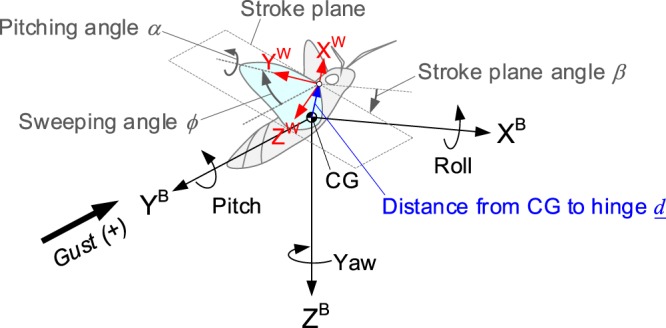
Kinematic definitions and the relation of two different coordinate systems.

We first planted the X-Y-Z axis on the center of gravity of the body, which are initially given as the forward, sideward (right), and downward directions, respectively, as the body-fixed coordinate system as shown in Fig. 10. Based on the previous study describing the wing kinematics of the hawkmoth with obeying 2–3–1–2 (or Y-Z-X-Y) set of Euler angles^15^, we then derived the rotation matrices in each component as follows:

1 $$\begin{array}{c}{{\bf{R}}}^{B\to W}={\bf{Y}}(\alpha ){\bf{X}}(\theta ){\bf{Z}}(\varphi ){\bf{Y}}(\beta )\\ \,\,\,=\,[\begin{array}{ccc}\cos \,\alpha  & 0 & -\sin \,\alpha \\ 0 & 1 & 0\\ \sin \,\alpha  & 0 & \cos \,\alpha \end{array}][\begin{array}{ccc}1 & 0 & 0\\ 0 & \cos \,\theta  & -\sin \,\theta \\ 0 & \sin \,\theta  & \cos \,\theta \end{array}][\begin{array}{ccc}\cos \,\varphi  & \sin \,\varphi  & 0\\ -\sin \,\varphi  & \cos \,\varphi  & 0\\ 0 & 0 & 1\end{array}][\begin{array}{ccc}\cos \,\beta  & 0 & \sin \,\beta \\ 0 & 1 & 0\\ -\sin \,\beta  & 0 & \cos \,\beta \end{array}],\end{array}$$

where the superscripts *B* and *W* on the Euler angle **R** indicate the body- and wing-fixed coordinate systems, and *β*, *ϕ*, *θ*, and *α* denote the stroke plane angle (constant), sweeping angle within the stroke plane, angle of deviation (zero in this study), and pitching angle, respectively. The Euler angle from the wing to body **R**^*W→B*^, which transforms the measurement values to the center of gravity of the body, can be obtained by inverting **R**^*B→W*^.

We had also used two individual vectors to transform the aerodynamic moment to the center of gravity. The first one ${\underline{d}}_{1}^{W}$ denotes the distance from the pivot point to the sensing point in the wing-fixed coordinate system. The second vector heading to the pivot point from the center of gravity ${\underline{d}}_{2}^{B}$ was written in the body-fixed coordinate system. The aerodynamic force/moment on the body then can be described as follows:

2 $${\underline{F}}^{B}={{\bf{R}}}^{W\to B}{\underline{F}}^{W}$$

3 $${\underline{M}}^{B}={{\bf{R}}}^{W\to B}({\underline{M}}^{W}+{\underline{d}}_{1}^{W}\times {\underline{F}}^{W})+{\underline{d}}_{2}^{B}\times {{\bf{R}}}^{W\to B}{\underline{F}}^{W},$$

where $${\underline{d}}_{1}^{W}={[\begin{array}{ccc}0 & 0.29b & 0\end{array}]}^{T}$$, and $${\underline{d}}_{2}^{B}={[\begin{array}{ccc}0.22b\sin \chi  & 0.12b & 0.22b\cos \chi \end{array}]}^{T}$$, and *χ* denotes an angle combined with a body angle in hover and a pivot angle from the longitudinal axis of the body, which were provided in the previous studies^15,16^.

Finally, the aerodynamic coefficients of lift, sideforce, and roll moment can be derived as follows:

4 $${C}_{L}=\frac{-2{F}_{Z}^{B}}{\rho {U}_{ref}^{2}S}=\frac{-2{F}_{Z}^{B}}{\rho {(2{\varphi }_{amp}fR)}^{2}{\hat{r}}_{2}^{2}S}$$

5 $${C}_{{F}_{Y}}^{B}=\frac{2{F}_{Y}^{B}}{\rho {(2{\varphi }_{amp}fR)}^{2}{\hat{r}}_{2}^{2}S}$$

6 $${C}_{{M}_{X}}^{B}=\frac{2{M}_{X}^{B}}{\rho {(2{\varphi }_{amp}fR)}^{2}{\hat{r}}_{2}^{2}S\bar{c}},$$

where ${\hat{r}}_{2}$ is non-dimensional second moment of the wing area^16^.

### Estimation *via* the quasi-steady aerodynamic model

The quasi-steady aerodynamic model, which had been built for hovering hawkmoth, had been employed to calculate the aerodynamic force/moment acting on the wings; accurate pitching moment estimation capability of this model delivers better performance in the flight stability analyses^25^. Empirically obtained aerodynamic force/moment coefficients and theoretically derived rotational lift and added mass^26,39^ had been employed, and the mathematical formulas in each model had been revised to improve an accuracy (refer to^26^ for details). Time-varying inflow velocities depending on the lateral gust had been also considered in this calculation. Each wing had been decomposed into 25 blade elements, which was also sufficient to converge the aerodynamic force/moment.
